# Identification of biomarkers, pathways and potential therapeutic agents for white adipocyte insulin resistance using bioinformatics analysis

**DOI:** 10.1080/21623945.2019.1649578

**Published:** 2019-08-13

**Authors:** Yemin Zhang, Yuyang Zheng, Yalin Fu, Changhua Wang

**Affiliations:** a Department of Pathology and Pathophysiology, Wuhan University School of Basic Medical Sciences, Wuhan, China; b Hubei Provincial Key Laboratory of Developmentally Originated Disease, Wuhan, China

**Keywords:** Insulin resistance, omental adipocyte, DEGs, hub gene, bioinformatics

## Abstract

For the better understanding of insulin resistance (IR), the molecular biomarkers in IR white adipocytes and its potential mechanism, we downloaded two mRNA expression profiles from Gene Expression Omnibus (GEO). The white adipocyte samples in two databases were collected from the human omental adipose tissue of IR obese (IRO) subjects and insulin-sensitive obese (ISO) subjects, respectively. We identified 86 differentially expressed genes (DEGs) between the IRO and ISO subjects using limma package in R software. Gene Set Enrichment Analysis (GSEA) provided evidence that the most gene sets enriched in kidney mesenchyme development in the ISO subjects, as compared with the IRO subjects. The Gene Ontology (GO) analysis indicated that the most significantly enriched in cellular response to interferon-gamma. The Kyoto Encyclopedia of Genes and Genomes (KEGG) pathway analysis revealed that the DEGs were most significantly enriched in cytokine-cytokine receptor interaction. Protein–Protein Interaction (PPI) network was performed with the STRING, and the top 10 hub genes were identified with the Cytohubba. CMap analysis found several small molecular compounds to reverse the altered DEGs, including dropropizine, aceclofenac, melatonin, and so on. Our outputs could empower the novel potential targets to treat omental white adipocyte insulin resistance, diabetes, and diabetes-related diseases.

## 1 Introduction

Insulin resistance and insulin resistance-related complication have become important causes of mortality and morbidity through the world. Many studies have ascribed insulin resistance and diabetes to obesity [1,2]. Obesity is broadly characterized as an expansion of white adipose tissue mass to reserve the excessive energy in the form of triglycerides. During the recent decades, white adipose tissue has been emerged as a metabolic regulator for its secreting adipokines including proinflammatory or anti-inflammatory factors [3]. One of the main reasons of dysfunction of white adipose tissue to the impaired suppression of lipolysis in the presence of high insulin levels, is white adipose insulin resistance that plays a critical role in the pathophysiology of diabetes, non-alcoholic fatty liver disease, diabetic cardiomyopathy and tumours [4–6].

Most previous studies have focused on the contrast of white adipose between the obesity and the lean [7–9]. However, not all obesity contributes to insulin resistance [10,11]. Hardy and his colleagues demonstrated not only that five genes including CCL2, CCL3, CCL4, CCL18 and IL8/CXCL8 were most highly expressed independent of body mass index (BMI) in the human omental adipose tissue of insulin-resistant obese (IRO) subject, as compared with insulin-sensitive obese (ISO) subjects, but that increased macrophage infiltration in the omental adipose tissue was correlated to insulin resistance. It was of great significance for their demonstration, however, the study only focused on that BMI-independent inflammation in omental adipose tissue associated with insulin resistance in morbid obesity [12].

In the present study, for the better understanding of the molecular biomarkers, the potential mechanisms and potential therapeutic agents for white adipocyte insulin resistance, diabetes, and other metabolic diseases, we downloaded two mRNA expression profiles from Gene Expression Omnibus (GEO, http://www.ncbi.nlm.nih.gov/geo/), which is an international public repository providing freely high-throughput microarray and relevant functional genomic data sets [13]. The total 30 samples of white adipocytes in two databases were collected from the human omental adipose tissue of IRO subjects and ISO subjects, respectively. With the performance of limma package in R software, 86 differentially expressed genes (DEG) which would be the novel diagnostic biomarkers, were screened between the IRO and ISO subjects. The potential mechanisms of obesity-induced insulin resistance such as kidney mesenchyme development, cellular response to interferon-gamma and cytokine-cytokine receptor interaction and so on were explored with the performance of Gene Set Enrichment Analysis (GSEA), the Gene Ontology (GO) analysis, the Kyoto Encyclopedia of Genes and Genomes (KEGG) pathway analysis and Protein–Protein Interaction (PPI) network. Ten hub genes (IL6, MMP9, CXCL8, CCL4, CXCL10, PTGS2, CCL2, SELE, CCL2, BCL2A1) were identified with the Cytohubba, including three genes (CCL2, CCL4, CXCL8) which had been identified in the previous study. CMap analysis was performed to discover several small molecular compounds to reverse the altered DEGs, including dropropizine, aceclofenac, melatonin, and so on. Our output could empower the novel and more comprehensive diagnostic and therapeutic targets for omental white adipocyte insulin resistance, and white adipocyte insulin resistance-induced diabetes and other chronic metabolic diseases.

## 2 Materials and methods

### 2.1 Microarray data archives

The expression profiles by an array of GSE15773 and GSE20950 were retrieved from GEO database. The samples in two databases were the human omental (for visceral) white adipocytes collected from insulin-resistant obese (IRO) and insulin-sensitive obese (ISO) subjects undergoing gastric bypass surgery between 2005 and 2009 at the University of Massachusetts Medical School [12]. GSE20950 collected 10 omental samples from IRO subjects and 10 omental samples from ISO subjects, and GSE15773 contained five IRO samples and five ISO samples. Totally, 15 omental samples from IRO subjects and 15 omental samples from ISO subjects. The statistical analyses for age, gender, height, weight, BMI, total cholesterol, high-density lipoprotein (HDL) cholesterol, low-density lipoprotein (LDL) cholesterol, triglycerides, and the number of lipids lowering therapy, between ISO and IRO subjects had no statistical significance. However, fasting glucose, fasting insulin and homeostatic model of assessment for insulin resistance (HOMA2-IR) between two groups had statistical significance [12]. The expression profilings of both databases were based on GPL570 (Affymetrix Human Genome U133 Plus 2.0 Array) platform. Series matrix files and data table header descriptions of two databases were downloaded from the GEO database to screen and verify hub genes involved in the IRO subjects.

### 2.2 Microarray data and degs identification

Following two databases annotated and consolidated by the performance of Perl script, sva package in R software (version 3.5.3) (University of California, Berkeley, CA) was applied for background expression value correction and data normalization [14]. DEGs with the threshold criterion of adjusted p < 0.05 and |log FC|; (fold change) >1 between the IRO and ISO subjects were screened in limma package in R software [15]. Pheatmap package in R software was subsequently performed to plot the heatmap of DEGs [16].

### 2.3 GO and pathway enrichment analyses

GO is a commonly used bioinformatic tool that provides comprehensive information on gene function of individual genomic products based on defined features. GO analysis of all detected genes was conducted by GSEA software (version 3.0) [17]. GSEA is a promising, widely used software package, which derives gene sets to determine different biological functions between two groups.

GO and KEGG pathway analyses of DEGs were performed via The Database for Annotation, Visualization, and Integrated Discovery (DAVID 6.8, http://david.ncifcrf.gov) [18]. The GO analysis consists of biological processes (BP), and cellular components (CC), molecular functions (MF). KEGG is a database resource for understanding high-level biological functions and utilities. Gene count >2 and p < 0.05 were set as the threshold.

### 2.4 PPI network creation and hub gene identification

PPI network of DEGs was constructed by Search Tool for the Retrieval of Interacting Genes (STRING10.5; https://string-db.org/) with a combined score >0.4 as the cut-off point [19]. Hub genes were identified using Cytohubba, a plug-in of Cytoscape software (Cytoscape, 3.7.1) and significant modules in the PPI network were identified by molecular complex detection (MCODE 1.5.1), another plug-in of Cytoscape software [20,21]. The parameters of DEGs clustering and scoring were set as follows: MCODE score ≥4, degree cut-off = 2, node score cut-off = 0.2, max depth = 100, and k-score = 2.

### 2.5 Correlation between hub genes and diabetes

Correlation between hub genes and diabetes was performed with the Attie Lab Diabetes database (http://diabetes.wisc.edu). The Attie Lab Diabetes database is a searchable resource of the gene expression data that is used to display the gene expression profiles of different experimental groups (lean and obese BTBR mice at 4 and 10 weeks of age) in any of six tissues, including adipose [22].

### 2.6 CMap analysis

The Connectivity Map (CMap) (https://portals.broadinstitute.org/cmap) is an open resource that links disease, genes, and drugs by similar or opposite gene expression profiles [23]. CMap analysis is used to predict potential small molecular compounds that can reverse altered expression of DEGs in cell lines. Mean < −0.4 and p < 0.05 were set as the screening criteria.

### 2.7 Statistical analysis

The statistical analyses of DEGs were done in R software. The p-values in GSEA analysis were analyzed with GSEA software (version 3.0). The p-value in the correlation between hub genes and diabetes were obtained from Attie Lab Diabetes database (http://diabetes.wisc.edu). The p-values in CMap analysis were analyzed in the CMap (https://portals.broadinstitute.org/cmap). Whenever asterisks are used to indicate statistical significance, *p < 0.05, **p < 0.01, and ***p < 0.001.

## 3 Result

### 3.1 Identification of DEGs related to insulin-resistant obese

**Figure 1.**
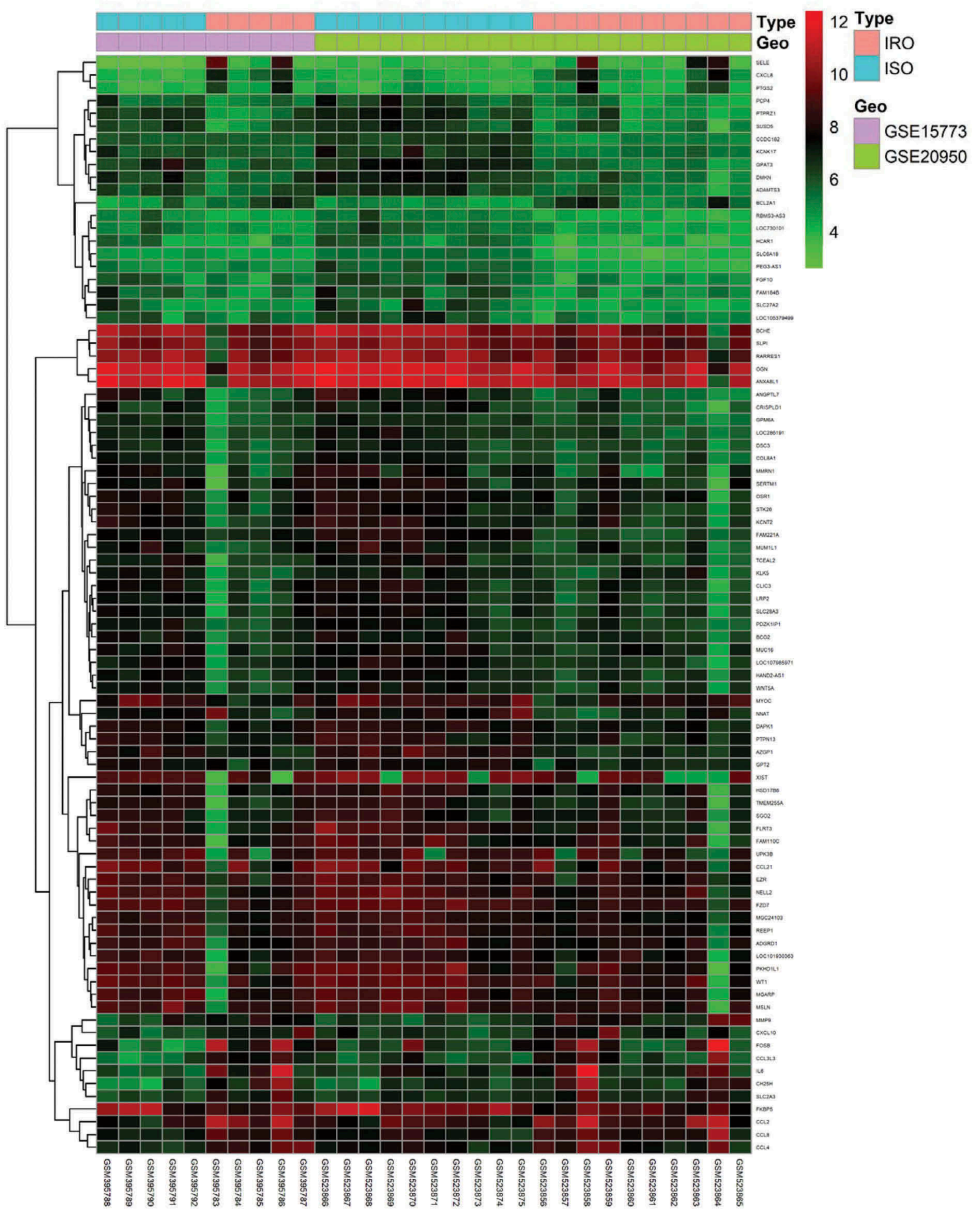
Heatmap of 86 DEGs screened by limma package in R software. Red areas represent highly expressed genes and green areas represent lowly expressed genes in omental adipose from IRO subjects compared with ISO subjects. DEG: differentially expressed gene; IRO: insulin-resistant obesity; ISO: insulin sensitivity obesity.

**Figure 2.**
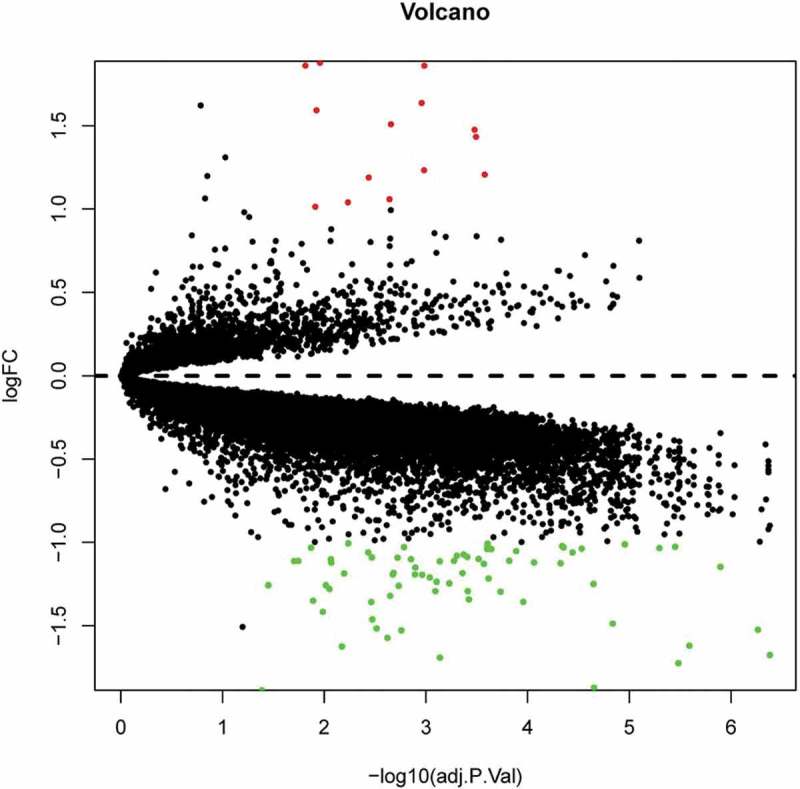
Volcano plot analysis identifies DEGs. Red dots represent 12 upregulated genes and green dots represent 64 downregulated genes in omental adipocyte from IRO subjects compared with ISO subjects.

To identify DEGs in the omental white adipocytes between ISO and IRO subjects, we retrieved relevant microarray expression profiles of GSE15773 and GSE20950 from GEO database. After consolidation and normalization of the microarray data, 86 DEGs between ISO and IRO subjects were screened by limma package (|logFC| >1, adjusted p < 0.05) as shown in the heatmap (Figure 1). Among them, 14 genes were upregulated and 72 genes were downregulated (Figure 2, Table 1).

**Table 1.** 86 differentially expressed genes (DEGs) between the IRO and ISO subjects.

Gene symbol	LogFC	P.Value	Adj.P.Val	Discription
SELE	1.879096	0.003469	0.010998	Selectin E
FOSB	1.862047	0.005321	0.015295	FosB Proto-Oncogene, AP-1 Transcription Factor Subunit
CH25H	1.861392	0.000147	0.001037	Cholesterol 25-Hydroxylase
CCL3L3	1.637939	0.000161	0.0011	C-C Motif Chemokine Ligand 3 Like 3
IL6	1.593556	0.003843	0.011902	Interleukin 6
CCL2	1.509892	0.000415	0.002205	C-C Motif Chemokine Ligand 2
CXCL8	1.476708	2.80E-05	0.000332	C-X-C Motif Chemokine Ligand 8
MMP9	1.434427	2.67E-05	0.000321	Matrix Metallopeptidase 9
CCL8	1.233571	0.000148	0.001041	C-C Motif Chemokine Ligand 8
BCL2A1	1.207528	1.96E-05	0.000264	BCL2 Related Protein A1
CCL4	1.189727	0.000822	0.003646	C-C Motif Chemokine Ligand 4
SLC2A3	1.059321	0.000437	0.002277	Solute Carrier Family 2 Member 3
CXCL10	1.040682	0.001537	0.005848	C-X-C Motif Chemokine Ligand 10
PTGS2	1.014023	0.003974	0.012219	Prostaglandin-Endoperoxide Synthase 2
EZR	−1.00561	0.001522	0.005807	Ezrin
FGF10	−1.00813	1.79E-05	0.000247	Fibroblast Growth Factor 10
LOC730101	−1.01143	7.46E-08	1.11E-05	Uncharacterized LOC730101
PDZK1IP1	−1.01724	1.80E-05	0.000248	PDZK1 Interacting Protein 1
GPT2	−1.02266	1.21E-06	4.58E-05	Glutamic–Pyruvic Transaminase 2
CCDC182	−1.02634	1.03E-08	3.54E-06	Coiled-Coil Domain Containing 182
LOC105379499	−1.02832	0.000278	0.001641	Uncharacterized LOC105379499
GPM6A	−1.03013	1.12E-06	4.42E-05	Glycoprotein M6A
RARRES1	−1.03198	0.004503	0.013463	Retinoic Acid Receptor Responder 1
RBMS3-AS3	−1.03424	1.97E-08	5.06E-06	RBMS3 Antisense RNA 3
DAPK1	−1.03754	5.38E-07	2.94E-05	Death Associated Protein Kinase 1
DSC3	−1.03987	1.58E-05	0.000227	Desmocollin 3
LOC286191	−1.03992	1.81E-05	0.000249	Uncharacterized LOC286191
MGC24103	−1.05252	6.67E-06	0.00013	uncharacterized MGC24103
OGN	−1.06052	0.000837	0.003696	Osteoglycin
PEG3-AS1	−1.06077	8.06E-07	3.62E-05	PEG3 Antisense RNA 1
BCO2	−1.07343	4.01E-05	0.000425	Beta-Carotene Oxygenase 2
WNT5A	−1.07968	5.03E-05	0.000497	Wnt Family Member 5A
PTPN13	−1.08707	3.56E-05	0.000392	Protein Tyrosine Phosphatase Non-Receptor Type 13
ADGRD1	−1.09049	0.000745	0.003394	Adhesion G Protein-Coupled Receptor D1
MUC16	−1.09179	0.000337	0.001897	Mucin 16, Cell Surface Associated
COL8A1	−1.0999	2.43E-05	0.000303	Collagen Type VIII Alpha 1 Chain
SGO2	−1.10134	0.002526	0.008593	Shugoshin 2
REEP1	−1.10205	0.000224	0.0014	Receptor Accessory Protein 1
HAND2-AS1	−1.11029	8.44E-06	0.000151	HAND2 Antisense RNA 1
FAM184B	−1.11123	5.55E-05	0.000529	Family With Sequence Similarity 184 Member B
SERTM1	−1.11179	0.006541	0.017993	Serine Rich And Transmembrane Domain Containing 1
HSD17B6	−1.11252	0.007312	0.019676	Hydroxysteroid 17-Beta Dehydrogenase 6
KLK5	−1.11374	8.95E-05	0.00073	Kallikrein Related Peptidase 5
ADAMTS3	−1.12108	3.45E-06	8.63E-05	ADAM Metallopeptidase With Thrombospondin Type 1 Motif 3
SLPI	−1.12146	0.002489	0.008497	Secretory Leukocyte Peptidase Inhibitor
KCNK17	−1.12528	1.29E-06	4.74E-05	Potassium Two Pore Domain Channel Subfamily K Member 17
FZD7	−1.12933	2.02E-05	0.000269	Frizzled Class Receptor 7
SLC6A18	−1.14746	1.11E-09	1.27E-06	Solute Carrier Family 6 Member 18
MYOC	−1.15129	0.000196	0.001269	Myocilin
OSR1	−1.18335	0.000381	0.002076	Odd-Skipped Related Transcription Factor 1
LOC107985971	−1.18454	4.19E-05	0.000436	Uncharacterized LOC107985971
LOC101930363	−1.18661	0.001718	0.006357	Uncharacterized LOC101930363
NNAT	−1.19253	0.000392	0.002116	Neuronatin
MUM1L1	−1.19258	0.000198	0.001281	Mutated Melanoma-Associated Antigen 1-Like Protein 1
CRISPLD1	−1.19426	0.000157	0.001088	Cysteine Rich Secretory Protein LCCL Domain Containing 1
SLC28A3	−1.21083	0.000124	0.000918	Solute Carrier Family 28 Member 3
PCP4	−1.21751	1.73E-05	0.000242	Purkinje Cell Protein 4
STK26	−1.23583	9.86E-05	0.000785	Serine/Threonine Kinase 26
LRP2	−1.24535	6.50E-05	0.000589	LDL Receptor Related Protein 2
HCAR1	−1.24879	3.40E-07	2.25E-05	Hydroxycarboxylic Acid Receptor 1
MSLN	−1.25677	0.002926	0.009622	Mesothelin
UPK3B	−1.2569	0.015278	0.035445	Uroplakin 3B
CLIC3	−1.2602	0.000327	0.001854	Chloride Intracellular Channel 3
CCL21	−1.27874	0.002635	0.00887	C-C Motif Chemokine Ligand 21
KCNT2	−1.29148	0.000103	0.000807	Potassium Sodium-Activated Channel Subfamily T Member 2
SUSD5	−1.29173	3.51E-05	0.000387	Sushi Domain Containing 5
NELL2	−1.29529	1.14E-05	0.000184	Neural EGFL Like 2
FKBP5	−1.31942	0.000426	0.002244	FKBP Prolyl Isomerase 5
SLC27A2	−1.34127	3.38E-05	0.000377	Solute Carrier Family 27 Member 2
FAM110C	−1.34917	0.004246	0.012852	Family With Sequence Similarity 110 Member C
TCEAL2	−1.35659	5.22E-06	0.00011	Transcription Elongation Factor A Like 2
MGARP	−1.3571	0.00076	0.003442	Mitochondria Localized Glutamic Acid Rich Protein
ANXA8L1	−1.41631	0.003195	0.010298	Annexin A8 Like 1
WT1	−1.46178	0.000737	0.003366	WT1 Transcription Factor
DMKN	−1.48658	1.41E-07	1.46E-05	Dermokine
BCHE	−1.51598	0.000649	0.003057	Butyrylcholinesterase
FAM221A	−1.52345	3.00E-10	5.43E-07	Family With Sequence Similarity 221 Member A
TMEM255A	−1.52749	0.0003	0.001738	Transmembrane Protein 255A
MMRN1	−1.57297	0.000467	0.002391	Multimerin 1
GPAT3	−1.61959	4.73E-09	2.57E-06	Glycerol-3-Phosphate Acyltransferase 3
PKHD1L1	−1.6242	0.001835	0.006696	Polycystic Kidney And Hepatic Disease 1 (Autosomal Recessive)-Like 1
AZGP1	−1.6757	3.82E-11	4.16E-07	Alpha-2-Glycoprotein 1, Zinc-Binding
FLRT3	−1.69116	8.87E-05	0.000727	Fibronectin Leucine Rich Transmembrane Protein 3
PTPRZ1	−1.72517	8.63E-09	3.29E-06	Protein Tyrosine Phosphatase Receptor Type Z1
ANGPTL7	−1.87141	3.28E-07	2.22E-05	Angiopoietin Like 7
XIST	−1.88809	0.018291	0.040896	X Inactive Specific Transcript

### 3.2 GO enrichment analysis of all detected genes

**Figure 3.**
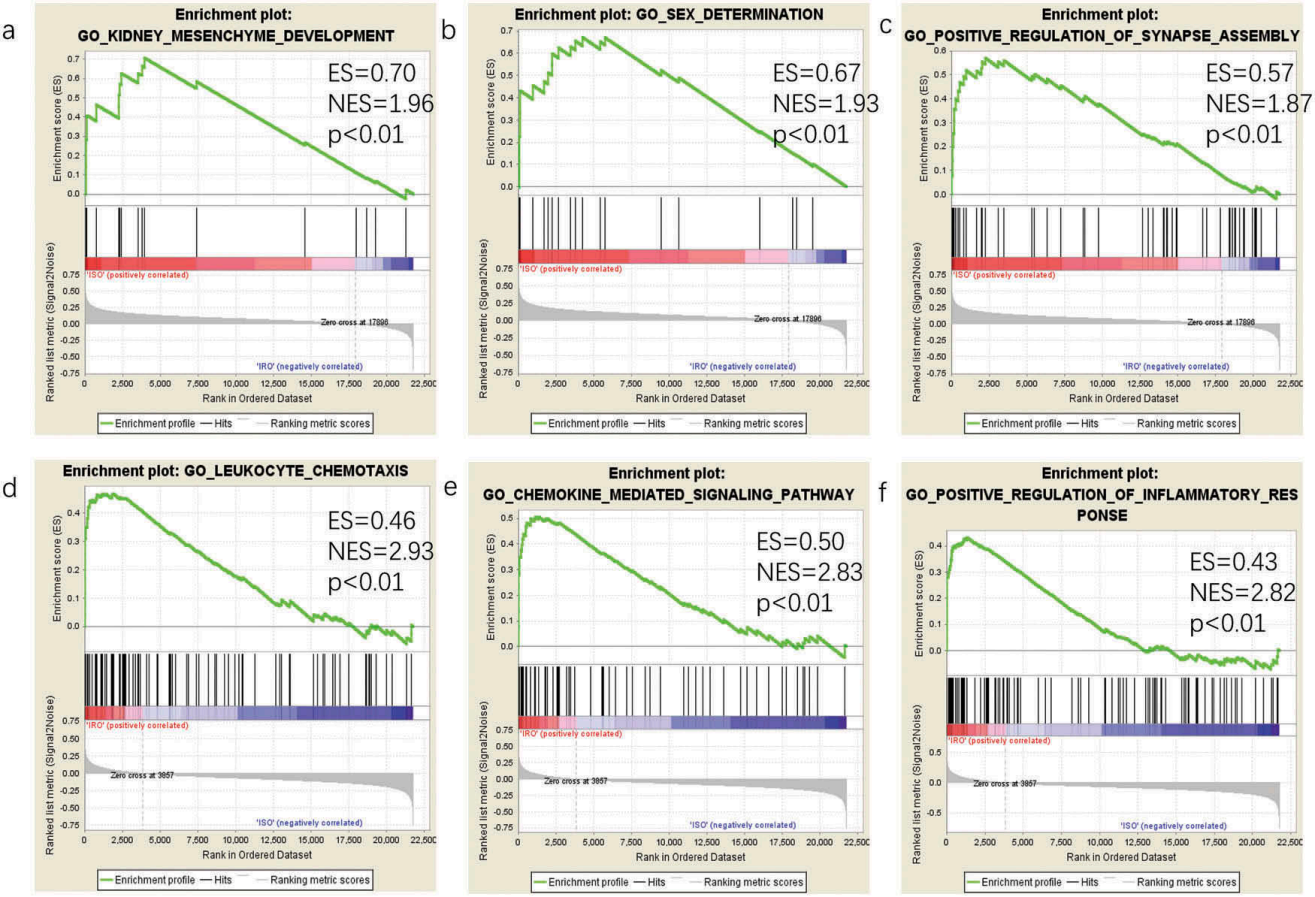
GSEA plot showing most enriched gene sets of all detected genes in the IRO subjects. The top-three most significant down-regulated enriched gene sets in the IRO subjects: kidney mesenchyme development (a), sex determination (b), positive regulation of synaspse assembly (c). The top-three most significant up-regulated enriched gene sets in the IRO subjects: leukocyte chemotaxis (d), chemokine-mediated signalling pathway (e), positive regulation of inflammatory response (f). GSEA: gene set enrichment analysis; NES: normalized enrichment score.

To identify gene sets with a statistically significant difference in the omental white adipocytes between ISO and IRO subjects, GSEA was performed, which showed most enriched gene sets of all detected genes in the IRO subjects. The top-three most significant-enriched gene sets negatively correlated with the IRO subjects were kidney mesenchyme development, sex determination, positive regulation of synapse assembly (Figure 3a–c), meanwhile, the top-three most significant-enriched gene sets positively correlated with the IRO subjects were leukocyte chemotaxis, chemokine-mediated signalling pathway, positive regulation of inflammatory response (Figure 3d–f).

### 3.3 GO enrichment analysis of DEGs

**Figure 4.**
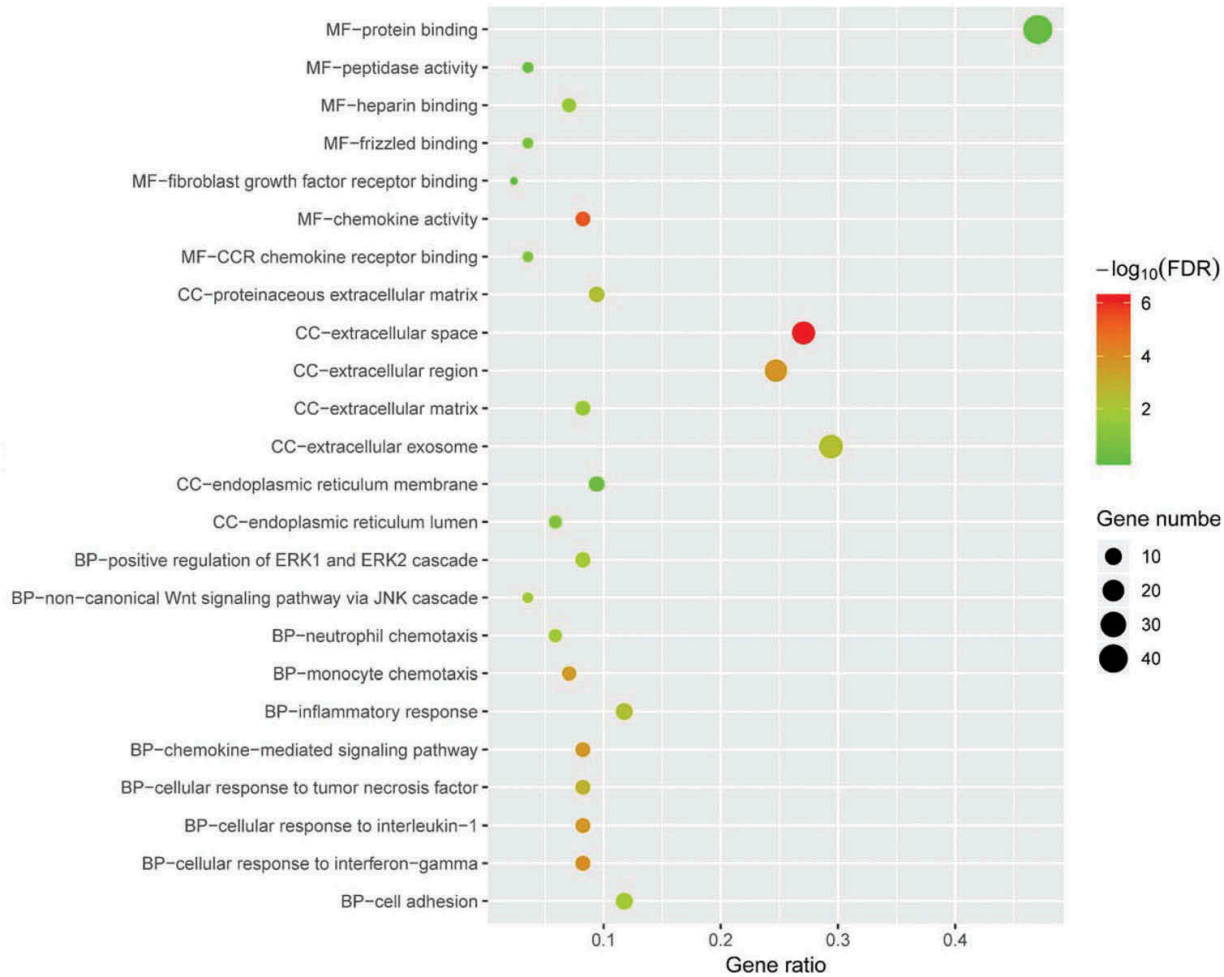
GO enrichment result of DEGs. The x-axis label represents gene ratio and y-axis label represents GO terms. The size of circle represents gene count. Different colour of circles represents different adjusted p value. DEG: differentially expressed gene; FDR: false discovery rate; GO: Gene Ontology.

To determine the biological features of DEGs, GO analysis was accomplished by DAVID online tools. The BP analysis revealed that the DEGs were major enriched in cellular response to interferon-gamma, chemokine-mediated signalling pathway, cellular response to interleukin-1, non-canonical Wnt signalling pathway via JNK cascade (Figure 4). The CC analysis showed that DEGs were enriched in extracellular space, extracellular region, extracellular exosome and proteinaceous extracellular matrix (Figure 4). Changes in MF of DEGs were significantly enriched in chemokine activity, heparin binding, protein binding, and peptidase activity (Figure 4).

### 3.4 KEGG enrichment analysis of DEGs

**Figure 5.**
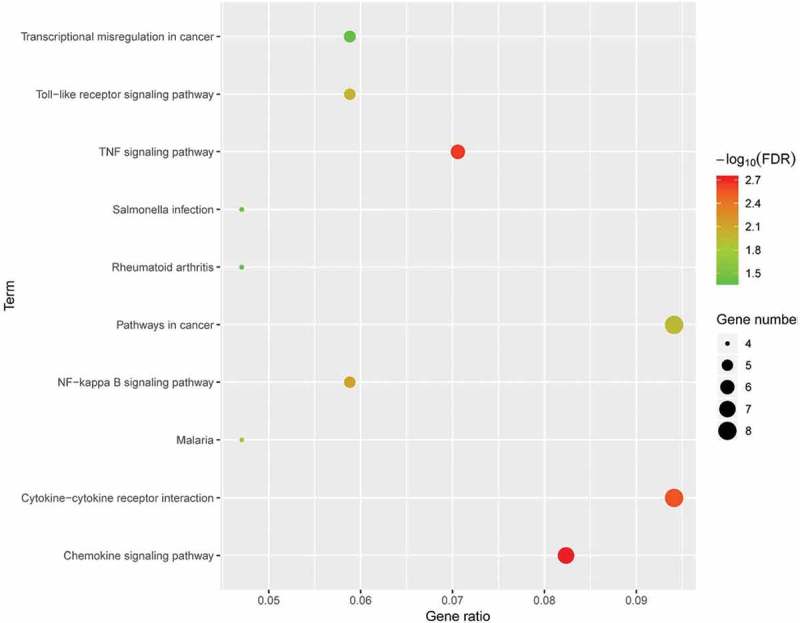
KEGG pathway analysis of differentially expressed genes. Advanced bubble chart shows enrichment of DEGs in signalling pathways. Y-axis label represents pathway, and X-axis label represents rich factor (rich factor = amount of DEGs enriched in the pathway/amount of all DEGs in background gene set). Size and colour of the bubble represent amount of DEGs enriched in pathway and enrichment significance, respectively. KEGG: Kyoto Encyclopedia of Genes and Genomes; DEG: differentially expressed gene; FDR: false discovery rate.

To explore the potential mechanism of these DEGs, KEGG pathway analysis was performed using DAVID online tools. The results of KEGG analysis revealed that DEGs were mainly involved in cytokine-cytokine receptor interaction, TNF signalling pathway, pathways in cancer, NF-kappa B signalling pathway (Figure 5).

### 3.5 PPI network analysis

**Figure 6.**
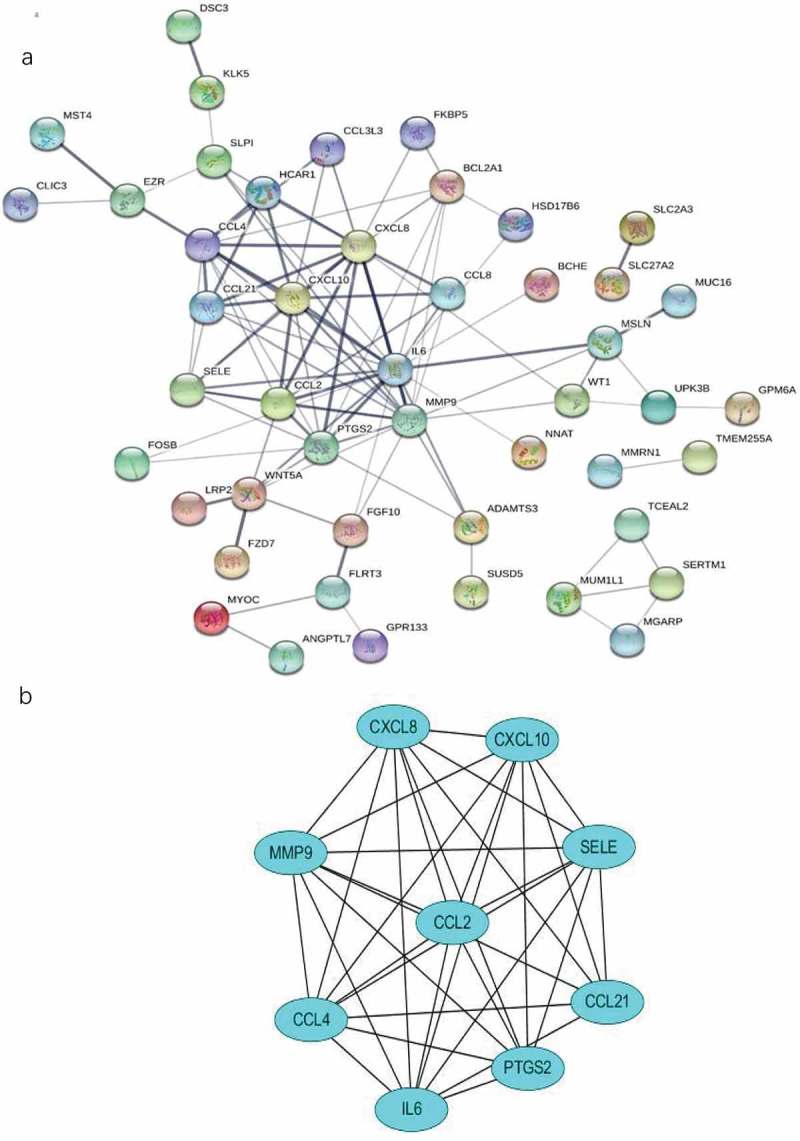
The PPI network and the most significant modules of DEGs. (a) The PPI network was analyzed by String software. Here were 47 nodes and 102 edged in the PPI network. (b) The most significant module identified by MCODE (score = 8.5). DEG: differentially expressed gene; PPI: protein–protein interaction.

To identify the most significant clusters of the DEGs, PPI network of DEGs was constituted by STRING. As shown in Figure 6(a), there were 47 nodes and 102 edged in the PPI network. The most significant modules (score = 8.5) were recognized by MCODE, a plug-in of Cytoscape. (Figure 6(b)).

### 3.6 Hub genes recognition

**Figure 7.**
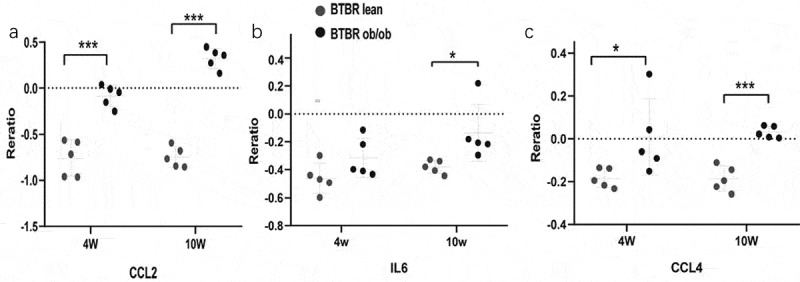
The expression of genes significantly upregulated in the adipose of the 10-weeks obese diabetic mice. (a) CCL2 gene expression was significantly upregulated in the adipocyte of the 10-weeks obese diabetic mice (p < 0.001). (b) IL6 gene expression was significantly upregulated in the adipocyte of the 10-weeks obese diabetic mice (p < 0.05). (c) CCL4 gene expression was significantly upregulated in the adipocyte of the 10-weeks obese diabetic mice (p < 0.001).

To identify the hub gene in the DEGs, Cytohubba, a plug-in Cytoscape was performed. All the gene code and edge were calculated. The top 10 genes were identified as hub genes (Table 2). To find the correlation between hub genes and diabetes, the Attie Lab Diabetes database was performed. BTBR mice become severely diabetic with obesity at 10 weeks of age. We checked the hub genes using the Attie Lab Diabetes database to identify the correlation between the hub genes and diabetes. We could find that the expression of CCL2, IL6, CCL4 were significantly upregulated in the 10-weeks BTBR obese diabetic mice (Figure 7).

**Table 2.** 10 hub genes identified by Cytohubba.

Gene symbol	Description	Degree of connectivity	logFC
IL6	Interleukin 6	20	1.59
MMP9	Matrix Metallopeptidase 9	16	1.43
CXCL8	C-X-C Motif Chemokine Ligand 8	15	1.47
CCL4	C-C Motif Chemokine Ligand 4	12	1.19
CXCL10	C-X-C Motif Chemokine Ligand 10	11	1.04
PTGS2	Prostaglandin-Endoperoxide Synthase 2	11	1.01
CCL2	C-C Motif Chemokine Ligand 2	9	1.51
SELE	Selectin E	8	1.88
CCL21	C-C Motif Chemokine Ligand 21	7	−1.28
BCL2A1	BCL2 Related Protein A1	7	1.21

### 3.7 CMap analysis

To search for potential small molecular compounds to reverse altered expression of DEGs, CMap analysis was performed. The most three significant small molecular compounds were dropropizine, aceclofenac, melatonin (Table 3).

**Table 3.** List of the 20 most significant small molecular compounds provided by CMap analysis to reverse altered expression of DEGs in cell lines.

CMap name	Mean	Enrichment	p	Percent non-null
Dropropizine	−0.741	−0.971	0.00167	100
Aceclofenac	−0.743	−0.965	0.00274	100
Melatonin	−0.731	−0.96	0.00352	100
Dihydroergotamine	−0.782	−0.958	0.00378	100
Levodopa	−0.708	−0.955	0.00429	100
Glycocholic acid	−0.702	−0.953	0.00467	100
Isocarboxazid	−0.688	−0.939	0.00767	100
Mafenide	−0.662	−0.932	0.00972	100
Methocarbamol	−0.723	−0.925	0.01153	100
Prestwick-1103	−0.648	−0.922	0.01223	100
Prednisone	−0.678	−0.918	0.01374	100
Withaferin A	−0.654	−0.907	0.01718	100
Pivampicillin	−0.633	−0.903	0.01871	100
Isoxicam	−0.643	−0.902	0.01911	100
Neomycin	−0.737	−0.9	0.02008	100
Niflumic acid	−0.669	−0.895	0.02185	100
Sulfacetamide	−0.642	−0.89	0.02416	100
Ethambutol	−0.617	−0.888	0.02491	100
Hesperetin	−0.589	−0.887	0.02527	100
Vinburnine	−0.627	−0.885	0.02648	100

## 4 Discussion

Insulin resistance is defined as the metabolic disordered situation that even higher concentration of insulin is insufficient to control the value of glycemia. During the recent decades, white adipose tissue has been emerged as an important regulator in the metabolism. Increasing studies have discovered that white adipose insulin resistance is strongly associated with the diabetes, cardiovascular diseases, and tumorigenesis [24–26]. Traditionally, white adipose includes subcutaneous adipose and visceral adipose. However, metabolic disorders are associated more strongly with visceral adiposity, rather than with subcutaneous adiposity [27]. The great concern is thus given to the diagnosis and therapeutic targets of visceral insulin resistance [28]. In the present study, bioinformatic methods are promising methods to analyze the critical genes and pathways which were associated with omental white adipose insulin resistance.

In the present study, a total of 21,755 genes were included. GSEA provided evidence that the most significant-enriched gene sets negatively correlated with the IRO subjects was kidney mesenchyme development. It has been discovered that BMP7, one of the gene ontology annotations in GO kidney mesenchyme development, could augment insulin sensitivity in mice with type 2 diabetes by potentiating PI3K/AKT pathway [29]. It will provide a new perspective on the therapeutic strategy on the insulin resistance and type 2 diabetes. Otherwise, GSEA provided further evidence that inflammation played a critical role in adipocyte insulin resistance, for the gene sets in GO that positively correlated with the IRO subjects were enriched in leukocyte chemotaxis and chemokine-mediated signalling pathway.

Based on the mRNA expression data, the 86 DEGs were identified between ISO and IRO groups. The analysis of BP in GO annotation indicated the DEGs were significantly enriched in cellular response to interferon-gamma, which was consistent with the previous demonstration that interferon-gamma released from omental adipose tissue of insulin-resistant humans impaired the response to insulin [30]. The most enriched gene set of DEGs in the BP of GO was inflammatory response, which was well consistent with the demonstration by Hardy and his colleagues [12]. The most gene set of DEGs in the CC of GO was enriched in extracellular exosome, which included 25 DEGs. Exosomes are extracellular microvesicles (30 to 150 nm in diameter) derived from various cells, transferring different proteins, non-coding RNA and coding RNA, which have been looked as diseases biomarkers or cell-cell communication factors [31,32]. Increasing studies have unveiled that exosomes derived from the insulin-resistant adipocyte were implicated in the skeletal muscle insulin resistance, obesity-related liver disease, atherosclerosis, and lung cancer [33–36]. Given the broad spectrum of the discoveries of the function of these exosomes, it is not surprising that exosomes derived from insulin-resistant adipocytes, functioned as independent metabolic units, which might provide a promising therapeutic target on the insulin resistance, diabetes, and related metabolic disorders [37]. The MF analysis of GO suggested that the DEGs were the most significant enriched in protein binding, suggesting that the interaction of two or more proteins played an important role in the adipocyte insulin resistance. Additionally, KEGG enrichment analysis of DEGs showed that these DEGs were mapped in cytokine-cytokine receptor interaction, TNF signalling pathway, toll-like receptor signalling pathway, pathways in cancer, all which were consistent with the previous demonstration that white adipocyte insulin resistance had cross-talking with inflammation and tumorigenesis [38,39].

In the present study, we found 10 hub genes including MMP9, IL6, CXCL8 (IL8), CCL4, CXCL10, PTGS2 (COX-2), CCL2 (MCP-1), SELE, CCL21 and BCL2A1. MMP9 has been reported to be positively correlated with omental adipocyte insulin resistance and MMP9 was decreased in response to pioglitazone [40,41]. Hoene et al. demonstrated that IL6 could induce insulin resistance and IL6 played a pivotal role in the metabolic process [42]. Kobashi and his colleagues suggested that IL-8 could induce insulin resistance via the inhibition of insulin-induced Akt phosphorylation in adipocytes [43]. Po-Shiuan et al. reported that COX-2 activation in visceral fat inflammation might crucially contribute to the development of insulin resistance and fatty liver in high-fat induced obese rats [44]. Kanda et al. demonstrated that abundance of MCP-1 mRNA in adipose tissue was increased in genetically obese diabetic (db/db) mice. Their research also revealed that insulin resistance induced by a high-fat diet was improved extensively in MCP-1 homozygous KO mice compared with WT animals and that acute expression of a dominant-negative mutant of MCP-1 ameliorated insulin resistance in db/db mice, which made it confirmed that MCP-1 played a critical role in adipocyte insulin resistance [45]. To the date, there is still no reports on the correlation between the genes of CCL4, CXCL10, SELE, BCL1A1 and CCL21 with adipocyte insulin resistance.

In the present study, we found several potential small molecular compounds to reverse the altered expression of the DEGs, which might improve white adipocyte insulin resistance. It was reported that the replacement therapy of melatonin might contribute to restore insulin resistance of cardiomyocytes and skeletal muscle [46–48]. Withaferin A also has been demonstrated that it played an important role on improving high-fat diet-induced obesity and palmitic acid-induced endothelial insulin resistance through attenuation of oxidative stress and inflammation [49,50]. Levodopa is known as a precursor to dopamine. The previous studies revealed that the altered dopamine turnover contributed to the behavioural disorders in brain insulin resistance, implying that dopamine might be a protective molecule [51]. However, whether dopamine and its precursor levodopa could improve adipocytes insulin resistance remains unclear. Yoshida et al. provided novel evidence that hesperetin directly inhibited TNF-alpha-stimulated FFA secretion to ameliorate FFA-induced insulin resistance in mice adipocytes [52]. However, the other small molecular compounds have not been reported to have the function to reverse insulin resistance or diabetes. All these small molecular compounds could be explored as the novel therapeutic targets to treat insulin resistance, diabetes, and related metabolic diseases.

In the present study, though we identified 10 hub genes of adipocyte insulin resistance and potential mechanism of white adipose insulin resistance with the bioinformatic analysis, further studies are urgently demanded to validate the hub genes, and further mechanisms would be uncovered. All the output will pave way to the potential therapeutic strategy to treat insulin resistance, diabetes and related metabolic disease.
